# Successful Pregnancy after Treatment with Chinese Herbal Medicine in a 43-Year-Old Woman with Diminished Ovarian Reserve and Multiple Uterus Fibrosis: A Case Report

**DOI:** 10.3390/medicines4010007

**Published:** 2017-02-09

**Authors:** Benqi Teng, Jie Peng, Madeleine Ong, Xianqin Qu

**Affiliations:** 1 School of Life Sciences, University of Technology Sydney, NSW 2007, Australia; tengbq@mail.sysu.edu.cn (B.T.); PengJie528@outlook.com (J.P.); Madeleine.L.Ong@student.uts.edu.au (M.O.); 2 Department of Obstetrics, The Third Affiliated Hospital of Sun Yat-sen University, 600 Tianhe Road, Guangzhou 510630, China; 3 Department of Gynaecology and Obstetrics, Suzhou Wuzhong People’s Hospital, Suzhou 215128, China

**Keywords:** Chinese herbal medicine, diminished ovarian reserve, infertility, in vitro fertilization, uterus fibrosis

## Abstract

**Objective:** To highlight a natural approach to coexisting oligomenorrhea, subfertility, luteal phase insufficiency and multiple fibroids cohesively when in vitro fertilisation (IVF) has failed. **Case Presentation:** A 43-year-old woman with diminished ovarian reserve and multiple uterine fibroids had previously been advised to discontinue IVF treatment. According to Chinese Medicine diagnosis, herbal formulae were prescribed for improving age-related ovarian insufficiency as well as to control the growth of fibroids. After 4 months of treatment, the patient’s menstrual cycle became regular and plasma progesterone one week after ovulation increased from 10.9 nmol/L to 44.9 nmol/L. After 6 months, she achieved a natural conception, resulting in a live birth of a healthy infant at an estimated gestational age of 40 weeks. **Conclusions:** The successful treatment with Chinese Herbal Medicine for this case highlights a natural therapy to manage infertility due to ovarian insufficiency and multiple fibroids after unsuccessful IVF outcome.

## 1. Introduction

Advanced maternal age contributes to subfertility due to diminished ovarian reserve (DOR) and decreased oocyte quality [1]. Reduced fertility potential may also be attributable to uterine fibroids which are more prevalent in women aged over 30 [2] depending on their size and location, which can affect embryo implantation [3]. Indeed, women with DOR and fibroids have a significantly low fertility rate despite advanced in vitro fertilization (IVF) techniques [4]. Given the limited treatment options after failure in IVF with DOR, these women often seek alternative therapy, such as Traditional Chinese medicine (TCM) to reserve adoption or using donor’s egg as the last option.

Chinese medicinal herbs have been used for more than two thousand years to treat gynaecological disorders including infertility. In the last decades, experimental and clinical studies have shown that these herbal ingredients can regulate gonadotropin-releasing hormone and ovarian sex hormone levels to induce ovulation and promote blood flow to the ovaries to improve ovarian reserve [5]. TCM is unique as it applies a formula with multiple natural ingredients that are capable of counteracting complex endocrine and reproductive disorders. Here, we present a successful live birth with Chinese herbal medicine (CHM) treatment to a 43-year-old nulliparous woman with DOR and uterus fibroids after failed conception from IVF.

## 2. Case Presentation

A 43-year-old woman visited our clinic for CHM treatment of infertility. She reached menarche at 13 years of age and at this time her menstrual cycle was regular with a normal menstrual flow. Oral contraceptive pills were taken from age 21–23 and she had no previous pregnancies prior to our treatment. She started trying to conceive at age 40 and her husband, aged 41, had no remarkable health problems and normal semen analysis as defined by the World Health Organization 2010 criteria [6]. After two years of trying to achieve natural conception, she was referred to a fertility specialist for IVF treatment. Prior to the initiation of treatment, a pelvic ultrasound showed that the right ovary was not visible and the left ovary measured 2.7 mL with only 3 antral follicles and both fallopian tubes were clearly patent. The uterus was bulky measuring at 103 × 125 × 68 mm with multiple fibroids including two larger subserosal fibroids. Serum anti-mullerian hormone (AMH) level of <3 pmol/L was within the <25% percentile for correlated age. Hormone testing on day 5 of her menstrual cycle identified follicle stimulating hormone (FSH) levels of 1.9 IU/L (normal range 1.5–10), luteinising hormone (LH) levels of 2.8 IU/L (normal range 2.0–12), Oestradiol (E2) levels of 913 pmol/L (normal range <320) and Progesterone (P) levels of <0.5 nmol/L (normal range 0.3–4.0). According to the ESHRE criteria this patient had suspected poor ovarian response [7]. Therefore, controlled ovarian hyperstimulation (COH) with daily FSH injections from day 3 of her cycle was applied for oocyte retrieval, however, only one mature oocyte was collected, and she failed to conceive after fertilized embryo transferring. The fertility specialist therefore advised her to seek egg donation or cease IVF. The patient consequently sought out treatment at our Chinese Medicine clinic in hope of achieving fertility.

At the time the patient visited the clinic her menstrual cycles ranged between 24 and 42 days. According to the diagnosis, basic formula containing 10 herbs was prescribed (Table 1), aiming to (1) improve diminished ovarian function and regulate the menstrual cycle and (2) restrain the growth of uterus fibroids which corresponds to tonifying vital essence and regulating and nourishing blood in TCM.

The herbal mixture was decocted into 500 mL and divided into two drinks daily. Ovulation was monitored by basal body temperature (BBT) and ovulation prediction kit. Plasma progesterone was measured one week of ovulation revealed by BBT chart and LH surge. Following initial consult and first month treatment with CHM, the patient underwent hormone testing one week after ovulation: P was 10.9 nmol/L, LH was 0.8 IU/L (normal range 2.0–12), FSH was 2.0 IU/L (normal range 1.5–10), E2 was 184 pmol/L (normal range 125–1300). After 12 weeks of treatment, the patient’s menstrual cycle became regular with cycle lengthen between 30 and 35 days. Pelvic ultrasound on day 9 of cycle indicated ovaries of both sides were normal in size and uterine size was reduced with measuring 84 × 62 × 56 mm as well as decreased sizes of intramural and subserosal fibroids. A blood test was also performed at day 23 of her menstrual cycle (about one week after ovulation) and revealed improvement of progesterone levels from 10.9 up to 28.1 nmol/L.

From week 13, the formula was slightly modified according to the follicular phase by adding Radix *Polygoni multiflori* (*He Shou Wu*) 10 g to nourish blood, and in the luteal phase by adding Radix Dipsaci (*Xu Duan*) 12 g and Cortex *Eucommiae ulmoidis* (*Du Zhong*) 9 g to assist in embryo implantation. Anti-fibrosis herb, *Prunellae vulgaris* and Rhizoma *Curcuma phaeocaulis* had been ceased after 4 months of treatment. After 5 months of taking CHM, although AMH was at 1.3 pmol/L, plasma progesterone levels after one week of ovulation went from 10.9 nmol/L to 44.9 nmol/L, suggesting that the quality of oocyte and function of corpus luteum had been improved. After 6 months of treatment with CHM, she achieved natural conception, resulting in a live birth of a healthy female infant weighing 3350 g at an estimated gestational age of 40 weeks through caesarean section. No modifications to the lifestyle of the patient were made during this period.

## 3. Discussion

The conventional approaches to sterility include ovulation induction, intrauterine insemination and IVF. In this case, woman with advanced maternal age, IVF may be the most successful technique to achieve conception. However, because of the DOR, this patient presented a very poor response to COH, resulting in unsuccessful IVF. Consequently, the patient turned to CHM. Our treatment achieved pregnancy and a healthy live birth with Chinese herbal formulae containing herbs supporting the folliculogenesis cycle, invigorating blood, improving microcirculation, and resolving masses to benefit both ovary and uterus.

Chinese herbal medicines have long been used for the treatment of infertility. Numerous studies demonstrated that CHM could regulate the gonadotropin-releasing hormone (GnRH) to induce ovulation and improve the uterus blood flow and menstrual changes of endometrium [10,11]. An advantage of CHM treatment is that it utilizes individualized formulas tailored to a patient’s condition. In this case, non-invasive treatment with CHM targeted two major causes underlying her infertility simultaneously, including poor quality of oocyte due to diminished ovarian reserve and uterine fibroids. The successful pregnancy eventually achieved through improvement of uterus environment and enhanced ovarian function.

It is generally accepted that submucousal fibroids but not subserosal fibroids have a negative impact on fertility by the virtue of their involvement in the endometrial cavity. This patient had multiple large subserosal and intramural fibroids of equal proportions. It has also been confirmed that myomas have higher levels of aromatase converting estrogens to estradiol, resulting in an imbalance of estradiol and progesterone, which is detrimental to embryo implantation and contributes to miscarriage [3]. In this infertility case, uterine fibroids have been considered as a treatment target because the estradiol levels of 913 pmol/L (normal range <320) was significantly high at follicular phase and there was also evidence of a bulky uterus caused by multiple fibroids. The large subserosal and intramural fibroids affect the endometrium homogeneous and may have negative impact on the embryo implantation on her natural conception and during IVF procedure. It is also a concern that these fibroids may aggressively grow during the pregnancy because of the ovarian hormone surge, subsequently affecting foetus development.

The herbal formula used in this case is able to balance *yin* and *yang*, nourish blood and invigorate blood circulation from a TCM perspective. In medical science, the CHM formula may restore ovarian function through improving blood flow to reproductive organs, regulating hormone secretions, lowering systemic inflammation as well as having anti-tumour properties. Among of them, Radix *Salvia miltiorrhizae*, Radix *Paeoniae lactiflorae*, Spica *Prunellae vulgaris* and Rhizoma *Curcuma phaeocaulis* have strong effects on blood stasis which is involved in mass (fibroids) formation in the reproductive system. Pharmacological studies have shown that these herbs reduce inflammation and inhibit neoplasia [12,13]. Meanwhile, the combination of Radix *Rehmannia glutinosa*, Semen *Cuscutae chinensis*, *Fructus mori*, *Fructus lycii*, Radix *Morindae officinalis* are commonly used in infertility due to ovarian factors, such as PCOS and primary ovarian insufficiency [13,14,15,16]. However, the precise mechanisms of Chinese herbs remain unclear with further research warranted.

After one month of treatment, day 6 oestradiol levels of 930 pmol/L reduced to 387 pmol/L. At the month five of the treatment, plasma progesterone from 10.9 nmol/L raised to 44.9 nmol/L, indicating improved ovarian function. Meanwhile, the size of the fibroids had shrunk with an overall reduced size of the uterus. These results indicate that CHM treatment inhibited fibroid growth and improved ovarian function, and may suggest enhanced quality of oocyte, evidenced by regularity of menstrual cycle, mid-cycle ovulation and elevated progesterone levels at luteal phase. Evidence suggests that oocyte quality profoundly affects fertilisation and embryo development [17]. The treatment achieved this woman’s natural pregnancy, suggesting the improvement of oocyte quality. The limitation in this case observation is that other assessments for oocyte quality such as morphological character of oocyte and corpus luteum and other biomarkers, such as mitochondrial status and glucose-6-phosphate dehydrogenase 1 activity and apoptosis of follicular cells, were not measured.

Infertility is a life-altering burden and the disorder affects a couple’s emotional health and wellness. Given the limited treatment options after failure in IVF, women with DOR who intend to get pregnant have to rely on assisted reproductive technology using donor eggs. The procedure is costly and only has a live birth rate of ~30% [4] and contributes to psychological distress. The results of this case demonstrate that CHM is capable of targeting multiple reproductive abnormalities involved in infertility. More specifically, herbal medicine can improve ovarian function and prevent inevitable miscarriage related to luteal phase defect and multiple fibroids. The successful treatment with CHM for this case highlights the potential of a natural approach to coexisting oligomenorrhea, subfertility, luteal phase insufficiency and multiple fibroids cohesively. CHM therapy offers a hope for aged women who have failed IVF cycles and decide to pursue parenthood with their own oocytes. The repeatability of CHM on infertility should be warranted through rigorously designed clinical trials.

## Figures and Tables

**Table 1:** Description of Chinese Herbal Formula and Possible Pharmacological Effects [8,9].

Herb Name	TCM Action	Possible Pharmacological Effects
*Shu Di Huang* (Radix *Rehmannia glutinosa*)	Tonifies Qi and Blood.	Improves blood supply to reproductive tissue.
*Dang Gui* (Radix *Angelicae sinensis*)	Tonifies and moves blood.	Improves blood supply to reproductive tissue and organs. Improves ovarian function.
*Tu Si Zi* (Semen *Cuscutae chinensis*)	Consolidates vital essence.	Promotes fertility by improving the quality of oocyte.
*Sang Shen Zi* (*Fructus mori*)	Strengthens vital essence.	Anti-oxidant and improves the quality of the oocyte. Anti-ageing properties.
*Gou Qi Zi* (*Fructus lycii*)	Nourishes blood.	Scavenges free radicals, increases DNA synthesis in reproductive tissue.
*Ba Ji Tian* (Radix *Morindae officinalis)*	Supports the vital essence.	Stimulates pituitary gland to improve ovulation.
*Dan Shen* (Radix *Salvia miltiorrhizae*)	Dissolves blood stasis.	Anti-inflammatory, promotes microcirculation to reproductive organs.
*Bai Shao* (Radix *Paeoniae lactiflorae*)	Nourish blood, regulate qi.	Anti-inflammatory.
*Xia Ku Cao* (Spica *Prunellae vulgaris*)	Dissolves mass formation.	Anti-fibrotic.
*E Zhu* (Rhizoma *Curcuma phaeocaulis*)	Dissolves blood stasis.	Anti-fibrotic.

Abbreviations: TCM: Traditional Chinese Medicine. Possible pharmacological effects were derived from.
